# Prevention of HIV-1 infection 2013: glimmers of hope

**DOI:** 10.7448/IAS.15.6.18066

**Published:** 2012-11-11

**Authors:** M Cohen

**Affiliations:** University of North Carolina School of Medicine, Department of Infectious Diseases, Chapel Hill, USA

## Abstract

The efficiency of transmission of HIV depends on the infectiousness of the index case and the susceptibility of those exposed. Infectiousness is dictated by the concentration of HIV-1 in relevant fluids (regardless of route of transmission) and the viral genotype and phenotype. People newly infected with HIV-1 (i.e. acute infection) and those with STI co-infections excrete such a large concentration of virus as to be “hyperinfectious.” The actual transmission of HIV likely occurs in the first few hours after exposure. The probability of transmission may be as low as 1/10,000 episodes of intercourse or 1/10 sexual exposures when anal intercourse is practiced. The transmission of HIV is generally limited to one or a small number of founder variants which themselves may be “hyperinfectious.” Synergistic behavioural and biologic HIV prevention strategies have been developed and implemented. Safer sex includes limiting the number of sexual partners, use of male latex condoms, and structural interventions to reduce exposure. These strategies appear to have contributed to reduced HIV incidence in many countries. Biological interventions have proved catalytic: these include treatment of inflammatory cofactors, voluntary male circumcision and use of antiviral agents either for infected people (who can be rendered remarkably less contagious) or as pre- and post-exposure prophylaxis (PrEP and PEP). Ecologic evidence suggests that broader, earlier antiviral treatment of HIV may be reducing incidence in some (but not all) populations. However, maximal benefit of HIV “treatment for prevention” and application of PrEP will likely require a program of universal “test and treat,” where many more infected patients are identified, linked to care, and treated very early in disease and for life. Community randomized trials designed to support this approach are under way in Africa. The “test and treat” prevention strategy is resource-intensive and serves to emphasize research that searches for a cure for HIV infection so that people living with HIV can eventually reduce or stop treatment. Likewise, success in HIV prevention emphasizes the importance of development of an HIV vaccine, which remains focused on agents that may evoke CTL responses, antibody dependent cytotoxicity, and (perhaps most important) broad neutralizing antibodies. A human clinical trial (RV144) and animal experiments have provided hope, excitement and a roadmap for development of an HIV vaccine.

